# Understanding the Role of Endocannabinoids in Posttraumatic Stress Disorder

**DOI:** 10.3390/ijms26125527

**Published:** 2025-06-09

**Authors:** Luke Ney

**Affiliations:** School of Psychology and Counselling, Queensland University of Technology, Brisbane 4059, Australia; luke.ney@qut.edu.au

**Keywords:** posttraumatic stress disorder, PTSD, endocannabinoids, cannabinoids, cannabis

## Abstract

Posttraumatic stress disorder is often treatment-resistant and recent research has suggested that treatment outcomes might be improved by modulation of the endocannabinoid system. The current review article describes animal and human research examining the effect of endocannabinoid modulation on posttraumatic symptoms, behaviours, and relevant memory processes. While the preclinical literature is reasonably consistent, emerging human literature is mixed. This review explores some potential reasons for why human research in this field is inconsistent and proposes multiple avenues for future research to answer these questions. Clinical trials testing the logistical challenges of cannabinoid administration and carefully designed human experimental studies are urgently required before cannabinoid therapy can be considered as an approach for treatment of posttraumatic stress disorder.

## 1. Introduction

Posttraumatic stress disorder (PTSD) is a global mental health concern [1,2]. While many people who experience a traumatic experience will recover favourably, a minority will develop PTSD, which is a chronic and debilitating illness that can last a lifetime [3,4,5]. Unfortunately, treatment for PTSD lags behind many other mental disorders, with available treatments effective in only about half of patients [6,7,8]. The research community has therefore committed significant resources to understanding how PTSD develops and how it may be effectively treated.

Within the past two decades, the endogenous cannabinoid (endocannabinoid) system has become a novel treatment target for PTSD [9]. The endocannabinoid system was earlier identified as a profuse lipid signalling system involved in many biological processes [10,11,12], including in the learning, memory, and stress domains that are relevant to PTSD [13,14,15,16]. Significant research attention has therefore focused on the possibility that, by leveraging the endocannabinoid system, we may be able to design more effective treatments for PTSD [17,18,19]. The field has recently begun to explore different treatment options for this endeavour [20], though clinical trials are still in their infancy. Regardless, many researchers in the field are optimistic that, by targeting the endocannabinoid system, we may be able to reduce the suffering associated with this disorder.

The potential role of endocannabinoids in fear and PTSD has now been explored for multiple decades [13,21]. How endocannabinoids control fear processes in the brain has been extensively modelled using preclinical methods and techniques, and it can no longer be debated that endocannabinoids have a large role in the expression of fearful behaviours that may relate to PTSD [22,23,24,25,26,27,28,29,30]. However, as the goal of this research is ultimately to reduce PTSD symptoms in humans, this review will focus largely on the existing human research that either reinforces or disputes this preclinical literature. This article will briefly review recent advances in our knowledge of how the endocannabinoid system regulates PTSD-like symptoms and fear conditioning in animal models, before comprehensively reviewing the literature exploring how endocannabinoids might be involved in human models of fear conditioning and human PTSD. This review will provide readers with an up-to-date explanation of the state of the PTSD–endocannabinoid literature, including future directions for where the field is moving next.

## 2. Background

### 2.1. Posttraumatic Stress Disorder

According to the Diagnostic and Statistical Manual 5th edition, PTSD is a chronic and debilitating disorder characterised by multiple symptom clusters, including negative changes to cognitions and mood, hyperarousal and hyperreactivity, avoidance behaviours, and intrusive memories of traumatic events [3,31]. Patients with PTSD often also suffer from poor-quality sleep and recurrent nightmares [32,33,34]. Additionally, while symptoms of PTSD can begin immediately after trauma exposure, a diagnosis can only be given if a minimum of one month has passed since the traumatic event [3]. This is because it is very normal to experience symptoms of PTSD in the immediate aftermath of trauma exposure; however, if symptoms continue long term (i.e., more than one month), then this indicates lack of recovery to normal functioning post-trauma [3]. Excessive PTSD symptomology occurring within one month following trauma exposure is classified as Acute Stress Disorder rather than PTSD [3], and the two conditions are considered to be separate disorders [35]. Moreover, in some cases, PTSD onset will occur several months or even years following trauma exposure [36]. The lifetime prevalence of PTSD worldwide is estimated to be an average of 3.9% [2], though it should be noted that this varies substantially depending on the country [1,2] as well as many other factors such as the type of trauma, the sex of the patient, the social environment, and the proximity to the traumatic event [37,38,39,40,41]. PTSD has a high economic and societal burden [42,43,44,45,46]. PTSD is also associated with high rates of partial disability among patients with the disorder [47,48,49,50]. Finally, PTSD is highly comorbid with other disorders, including major depressive disorder, anxiety, and substance use disorders [51,52,53], as well as suicide [54]. Therefore, the imperative to improve treatment outcomes in PTSD has become a global priority within mental health research.

### 2.2. How Is PTSD Studied Experimentally?

Experimental studies have played a huge role in understanding the mechanisms underlying PTSD and how treatments may be improved [55]. In PTSD, several experimental paradigms have had a significant influence. This section will briefly review the most influential paradigms, as well as those which are most relevant to the current review. Readers should be aware that other paradigms to understand PTSD exist but are not covered in this review. Some other notable paradigms include variations of the classical odd-ball task [56] and the dot-probe task, which measure attentional bias to emotional stimuli [57]. As these types of paradigms have very rarely been explored in the context of endocannabinoids, they will not be reviewed in this article.

#### 2.2.1. Fear Conditioning

The paradigm that has arguably been the most influential in understanding PTSD is fear conditioning [58,59,60,61,62]. Fear conditioning is a paradigm that examines learning associations between neutral stimuli (called conditioned stimuli, or CS+) and an aversive outcome (called the US, e.g., an electric shock). Repeated pairing between the CS+ and US results in the previously neutral stimulus taking on a negative valence, as subjects learn that the CS+ predicts an aversive outcome. This learning process mimics fear learning in PTSD, where repeated (or one-time) exposure to trauma creates a threatening memory of objects and people in the surrounding environment [63,64]. Fear conditioning paradigms also involve extinction learning, during which the CS+ is presented repeatedly without aversive reinforcement. This phase of a fear conditioning experiment is designed to emulate the memory process involved in exposure therapy, where a safety memory is created through repeated exposure to the fear stimuli and which later competes with and inhibits the fear memory [65,66,67,68]. In humans, outcome measures typically involve psychophysiological measures, such as skin conductance responding, neural imaging, and subjective reports of US expectancy and valence [69]. In animals, outcome measures typically involve behavioural evaluation, such as rate of freezing of the animal or activity suppression [70]. The long-term efficacy of extinction learning can later be modelled using return of fear manipulations in the laboratory [61,71]. These manipulations include renewal (a change in context from extinction back to the original acquisition context during a subsequent renewal phase), reinstatement (where the US is presented alone after extinction), spontaneous recovery (where the extinction phase is presented again after a passage of time), and re-acquisition (where the US is paired with the CS+ again after extinction).

Although valid criticisms of fear conditioning exist [72,73,74], the study of extinction in particular has had substantial clinical relevance for PTSD [75,76]. In particular, this paradigm has assisted researchers in understanding many nuances of the memory mechanisms underlying recovery from disorders such as PTSD [65,77,78,79], and with this improved knowledge, it is hoped that psychological treatments can be finetuned to improve patient outcomes. Another benefit of the fear conditioning paradigm is that, in comparison to some other paradigms such as trauma film (reviewed below), there are roughly analogous experiments available between human and animal subjects [80]. This means that invasive and precise experiments can be conducted in animals and later translated to human subjects. In the case of endocannabinoids, animal models of fear conditioning have been essential to understanding how specific processes within conditioning are influenced by endocannabinoid signalling. This review will aim to concisely summarise this literature in Section 3.1.

#### 2.2.2. Trauma Film Paradigm

While fear conditioning is capable of closely examining the discrete learning and memory mechanisms believed to underlie PTSD, the stimuli that are used are typically basic and may not be valid for exploring the complexity of real-world trauma. To better mimic traumatic experiences in the laboratory, the trauma film paradigm was developed in the 1960s [81,82]. This paradigm involves the presentation of a traumatic video with depictions of death or serious injury, followed by the completion of subjective reports of intrusive memory diaries for up to a week after the laboratory session [83]. This paradigm is obviously not suitable for animals, as animals will neither watch the trauma film nor provide subjective reports of intrusive memories. However, in humans this paradigm has proven to be a close analogue to real traumatic experience and post-trauma intrusive symptomology, and has resulted in the development of new potential interventions for PTSD [84,85].

Holmes and Bourne [86] explain that the use of perceptual priming elements observed during intrusive memory reporting in the trauma film paradigm align with the cognitive model of PTSD [63]. Research has subsequently explored how various manipulations post film-viewing may reduce the frequency and intensity of distressing film-related intrusions [87,88]. One especially interesting finding has been that the interruption of perceptual priming memory processes caused by engagement in visuospatial working memory tasks (i.e., Tetris) reduced intrusive memories in the laboratory [84], and this approach has subsequently been successfully tested in clinical trials [89,90,91,92]. The paradigm, therefore, has good translational value. Extensions of the fear conditioning paradigm (replacing shock USs with film clip USs) has produced an additional research tool that has begun to be explored in the field [93,94].

#### 2.2.3. Additional Animal Models of PTSD

While fear conditioning and the trauma film paradigm are the dominant PTSD experimental paradigms used in humans, preclinical researchers have additionally used different models to explore the processes by which symptoms arise and are regulated in PTSD [95,96]. These paradigms typically involve the introduction of a significant stressor, after which changes to animal behaviour and biology can be observed.

The single-prolonged stress procedure involves the prolonged forced restraint of the (typically rodent) animal, followed by a forced swim test. These stressors are classified as physical stressors. By comparison, models involving social stress are frequently used. These may include, but are not limited to, social isolation, early life stress (such as maternal isolation), and social defeat by an aggressive animal [97]. Psychological stress paradigms involve models such as predator-based psychosocial stress and predator scent stress, where the animal is exposed directly to a predator or to the predator’s scent (e.g., a rat is exposed to a cat’s scent) [98,99,100]. Animal stress may also be induced during tasks such as the open field task [101] and paradigms involving repeated and/or chronic exposure to the stressors mentioned above.

Since PTSD is often conceptualised as a disorder relating to memory [102], the above paradigms are often complemented by memory paradigms. These include, but are not limited to, spatial memory-based tasks such as the radial arm maze [103] and the Morris water maze [104], object recognition tasks [105], and fear conditioning (including inhibitory avoidance) [106].

While human studies often rely on producing outcome measures such as psychophysiological recordings and subjective participant responses [60,107,108], animal models usually rely on behavioural outcomes, such as frequency of freezing behaviours, social behaviour, and evidence of memory during memory tasks.

### 2.3. Endocannabinoid System Primer

**Figure 1:**
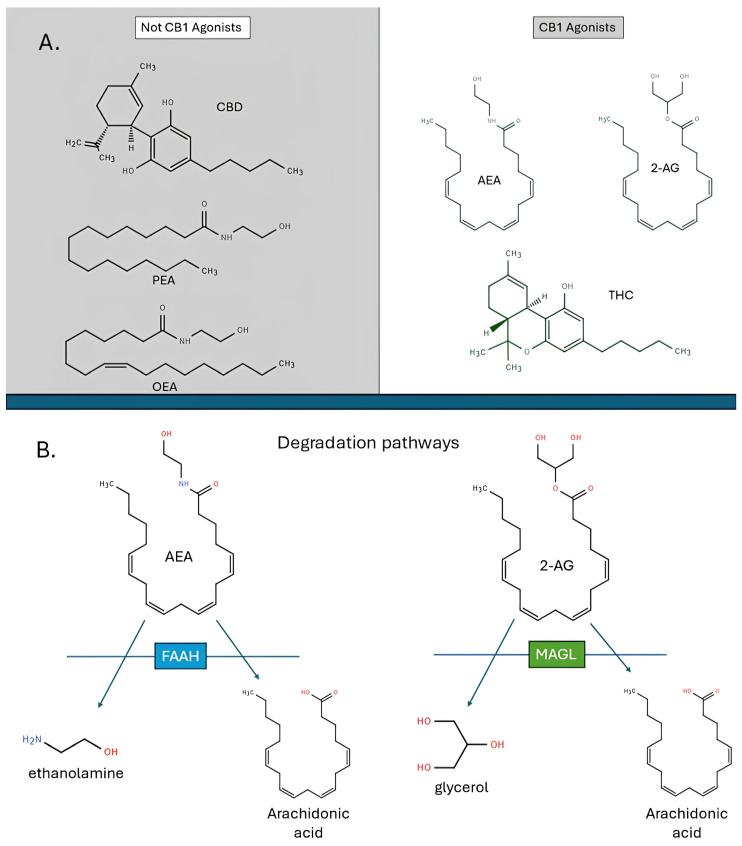
CB1 agonists (**A**) and degradation pathways (**B**) discussed in the current review. 2-AG = 2-arachidonoyl glycerol, AEA = arachidonoyl ethanolamide, CBD = cannabidiol, FAAH = fatty acid amide hydrolase, MAGL = monoacylglycerol lipase, OEA = oleoylethanolamide, PEA = palmitoylethanolamide, THC = delta9-tetrahydrocannabinol.

The endocannabinoid system is a lipid signalling system that was identified following the synthesis and characterisation of the active constitute of cannabis [10,109,110,111,112]. The initial breakthroughs came with the discovery and cloning of the cannabinoid receptors [109,113]. Subsequent major breakthroughs occurred with the identification of arachidonyl ethanolamide (AEA) [114] and 2-arachidonyl glycerol (2-AG) [115], which are now known to be endogenous ligands that bind directly to cannabinoid receptors 1 (CB1, primarily in the central nervous system) and 2 (CB2, primarily in the peripheral nervous system) [10,116]. Other endocannabinoid-like molecules, such as palmitoylethanolamide (PEA) and oleoylethanolamide (OEA) are considered part of the expanded endocannabinoid system, but do not bind directly to cannabinoid receptors [11]. The enzymes fatty acid amide hydrolase (FAAH) and monoacylglycerol lipase (MAGL) are primarily responsible for the hydrolysis of AEA and degradation of 2-AG, respectively (Figure 1). Higher expression of FAAH is associated with lower AEA (as well as OEA and PEA) levels, whereas higher expression of MAGL is associated with lower 2-AG and related glycerol levels. FAAH inhibition involves the administration of a drug that selectively impairs the synthesis of the FAAH enzyme, resulting in higher levels of AEA due to reduced FAAH activity. Similarly, MAGL inhibition involves impairing MAGL synthesis to increase 2-AG levels. By comparison, delta9-tetrahydrocannabinol (THC) is a direct agonist of CB1 receptors [117] and CBD is believed to be an indirect agonist of CB1 via FAAH inhibition [118]. While THC produces psychotropic effects, CBD is non-psychoactive.

The implications of the discovery of the endocannabinoid system were profound, but eventually realised to be understated, given evidence of the immense profuseness of the endocannabinoid system through both the central and peripheral nervous systems [10,119]. Shortly after the system’s discovery, it was realised that the endocannabinoid ligands acted as secondary messengers, were synthesised on demand, and, using these characteristics, were involved in the regulation of a large proportion of biological processes in the body, including fundamental synaptic processes such as long-term depression and potentiation [11,120,121,122,123,124,125]. It was quickly surmised that the endocannabinoid system, via the mediation of other biological systems, must be involved in many processes relevant to human health [126].

In particular, the role of endocannabinoids in response to stress was identified [127], with preclinical evidence showing that AEA and 2-AG play critical roles in the regulation of the glucocorticoid response to stress [128,129,130,131,132]. While relevant to PTSD, this literature has been comprehensively elsewhere and will not be re-reviewed extensively in this article. Readers may refer to any one of the other excellent review articles on this topic [127,133,134].

## 3. Understanding Endocannabinoid Involvement in PTSD Using Preclinical Models

Most of our knowledge of how the endocannabinoid system may be involved in fear conditioning and PTSD has been developed using preclinical experimental models. This section will review the research testing the role of cannabinoids and endocannabinoids in animal models of stress (Section 3.1), fear conditioning (Section 3.2), and in PTSD-like symptomology and behaviours (Section 3.3). Readers should be aware that the focus of this review is chiefly on translational (i.e., human) research, therefore the sections below are not comprehensive but only reflect the overall knowledge and recent advancements in the field.

### 3.1. Animal Models of Endocannabinoid Modulation of Stress

Seminal research in the early 2000s revealed that, in vitro, the glucocorticoid-based inhibition of paraventricular nucleus neurons and the subsequent inhibition of corticotropin-releasing hormone synthesis depended on retrograde endocannabinoid signalling [135,136]. Research in the early 2000s also found that corticotropin-releasing hormone was found to be highly co-expressed with CB1 receptors in the forebrain [137]. It was, therefore, suspected that endocannabinoids could be involved in the regulation of the hypothalamic–pituitary–adrenal (HPA) axis response to stress [138]. Subsequent research confirmed this hypothesis, as it was discovered that a reduction in AEA in the basolateral amygdala is required for HPA axis activation, suggesting that AEA tonically gates the HPA response [129,139]. Research further explored the mechanisms involved in HPA gating by AEA and found that CRH-mediated increases in FAAH expression is responsible for the decrease in amygdala AEA prior to HPA onset [140,141,142].

While AEA is believed to tonically gate the HPA stress response, 2-AG is believed to be involved in the termination of the response through the facilitation of glucocorticoid-mediated negative feedback [135,143,144]. Research that supports this hypothesis has shown that 2-AG levels increase in the hippocampus 30 to 60 min following stress, while the inhibition of CB1 in the frontal areas of the brain prevents negative feedback of the HPA axis [128,145,146,147,148]. Likewise, the inhibition of MAGL, but not FAAH, maintains increased corticosterone expression after stress onset [149,150,151]. Given the evidence described above, it has therefore been widely hypothesised that endocannabinoids are involved in both the initiation and termination of the HPA axis response to stress [127,133,134].

### 3.2. Animal Models of Fear Conditioning

#### 3.2.1. Seminal Fear Conditioning Endocannabinoids Research in Animals

The first evidence that endocannabinoids were involved in fear conditioning was published shortly after the discovery of the endocannabinoid ligands. Marsicano, Wotjak, Azad, Bisognok, Rammes, Casciok, Hermann, Tang, Hofmann, Zieglgansberger, Di Marzok, and Lutz [13] reported that both CB1-deficient mice and mice subjected to pharmacological CB1 antagonism had reduced extinction learning and retention compared to controls. Subsequent studies were able to replicate this effect, with CB1 antagonism and/or genetic CB1 knockout associated with significantly impaired retention of extinction learning during standardised preclinical fear conditioning experiments [152,153,154,155]. Isolating a role of the endocannabinoid ligands in this process, studies also found that fear extinction retention was also improved by the pharmacological inhibition of FAAH, diacylglycerol lipase (DAGL), and MAGL [152,153,156,157], though most studies have focused on FAAH inhibition. This effect is reversed by concurrent CB1 but not necessarily CB2 antagonism, suggesting a critical role of the CB1 receptor specifically [158,159,160]. The pharmacological agonism of CB1 receptors has similarly been shown to improve fear extinction learning and the retention of extinction memories [161,162,163,164,165,166].

Studies have shown that both THC and CBD can also improve fear extinction, which is an effect that is also moderated by the CB1 receptor, and potentially additionally the CB2 receptor in the case of CBD [167,168,169,170,171,172,173]. Moreover, while most studies have focused on AEA and FAAH, research also suggests that the augmentation of 2-AG can reduce conditioned avoidance behaviour and improve extinction [174,175], though this effect is region-specific, with higher 2-AG in the amygdala and hippocampus associated with impaired extinction [176,177,178]. As described in Section 3.2.3., this result is also not always replicated, with many studies finding that 2-AG and MAGL inhibition can conversely enhance fear expression and impair extinction [175,177,179,180]. The shock and reminder model of PTSD was used to show that FAAH inhibition was superior to direct CB1 agonism in reducing PTSD-like symptoms, though both CB1 agonism and FAAH inhibition were again CB1-dependent [181].

#### 3.2.2. Fear Conditioning Findings with High Relevance to Therapy

Critically, preclinical research has begun to also demonstrate important nuances in how endocannabinoid signalling affects fear conditioning, which may have clinical relevance for treatment in humans. For example, while endocannabinoid signalling has been shown to be essential to extinction learning, Morena et al. [182] found that FAAH inhibition had no effect on freezing behaviours if it was administered without concurrent extinction training, suggesting that the beneficial effects of endocannabinoid modulation on fear behaviours depends on active engagement in extinction learning or exposure therapy. Moreover, Molla et al. [183] reported that behavioural effects of increasing 2-AG and AEA levels in the prefrontal cortex during fear conditioning did not occur in rodents until late adolescence, suggesting important moderating effects of age on the efficacy of endocannabinoid-related benefits during fear conditioning. It is likewise known that endocannabinoid levels both in the brain (e.g., prefrontal cortex) and in circulating serum increase significantly with older age [184,185,186]. This suggests that cannabinoid administration should be weighed against the age of the patient undergoing exposure therapy. It is also important to emphasise that different brain regions recruit endocannabinoid signalling differently—for example, one study found that rodents that were resilient to fear generalisation had significantly elevated 2-AG levels in the prelimbic cortex but reduced 2-AG levels in the ventral hippocampus [187].

Finally, the effects of endocannabinoids on fear conditioning have been reported to be sex-dependent [22,168,188,189]. This is potentially important, particularly given evidence that fear conditioning is affected by sex [190,191] and that PTSD is significantly more prevalent in females compared to males [192,193]. However, the directionality of sex-dimorphic endocannabinoid effects on fear conditioning are not yet clear. For example, while Mizuno, Matsuda, Tohyama, and Mizutani [179] found that direct CB1 agonism affected fear retrieval in both male and female rats, MAGL inhibition was only effective in female rats, and CB1 antagonism was ineffective in both sexes. By comparison, Morena et al. [194] reported that increasing 2-AG resulted in higher active fear responses and increasing AEA resulted in impaired fear extinction in female rats, with no effect in male rats. Sex differences in cannabinoid metabolism and subjective effects have also been reported in human subjects [188,195,196,197,198,199], suggesting that endocannabinoid effects on fear conditioning should be expected to be sex-dimorphic. Although fear conditioning results are not particularly consistent at this stage, endocannabinoid modulation may produce substantially different effects on males compared to females during fear conditioning, which may extend to human exposure therapy. Understanding the role of sex in this field may be important, given the prominent sex differences in PTSD diagnosis and symptom severity, where females are twice as likely to suffer from PTSD even after controlling for trauma type [1,200,201,202,203].

#### 3.2.3. Discrepant Findings

The above sections describe evidence supporting a role of the endocannabinoid system in preclinical fear conditioning. It is important to note, however, that not all studies have replicated the findings described above. For example, the systemic administration of the MAGL inhibitor JZL184 in mice was found to impair short-term fear extinction in one study [175] and enhance fear expression or reduce extinction retention in other studies [177,179]. Likewise, some research has found evidence that FAAH inhibition can produce either no effect [179,180,182] or an impairing effect on fear extinction and extinction retention [194]. Therefore, not all studies have supported a role for endocannabinoids in reducing fear in animals.

#### 3.2.4. Subsection Summary

Overall, these studies provide support for the hypothesis that the endocannabinoid system is involved in preclinical fear conditioning and provide many potential pharmacological tools by which fear conditioning might be modified in humans, including via FAAH or MAGL inhibition, or direct intervention with THC or CBD. In the next section, the role of endocannabinoid signalling in animal models of PTSD that do not rely on traditional conditioning paradigms will be reviewed.

### 3.3. Animal Models of PTSD

As described above, there are many other preclinical paradigms that are associated with PTSD symptomology aside from fear conditioning. This section will briefly review how these paradigms have been used to explore the role of the endocannabinoid systems in PTSD.

#### 3.3.1. General Findings

Aligning with findings from the fear conditioning literature, FAAH inhibition has been found to reduce anxiety behaviours that develop as a result of exposure to single prolonged stress and predator scent exposure [204,205,206]. The mechanism responsible for this effect has been reported to be CB1-dependent and associated with AEA elevation after FAAH inhibition [207]. Other studies have also found that direct CB1 agonism reduces PTSD-like behaviour following exposure to significant stressors [208,209]. CBD has also been reported to reduce PTSD-like symptoms, though the mechanisms are proposed to differ from conventional CB1 agonists [210,211,212,213]. 

While the effects of cannabinoids and endocannabinoids have largely been found to be CB1-dependent, there are multiple other studies that have reported similar interactions and dependency with other biological systems. For example, decreases in anxious and depressive behaviours are found to be dependent on β-catenin in the nucleus accumbens [214]. It is also very likely that the endocannabinoid system interacts with the glucocorticoid [133,134,215,216,217] and dopaminergic [218,219,220] systems, as well as others [166,221,222,223], to produce its effects.

#### 3.3.2. Sex Differences

Interestingly, there are some studies that have examined sex effects in PTSD symptomology preclinical models, and the findings from these studies are somewhat aligned with the fear conditioning literature. Firstly, predator scent stress increased female, but not male, levels of AEA in the central amygdala, and decreased basolateral amygdala levels of 2-AG in female, but not male, rats [224]. Conversely, swim stress significantly increased male but not female amygdala 2-AG levels in another study [225]. Moreover, CBD was reported to be only effective in producing anxiolytic effects in female rats exposed to the elevated plus maze during the late diestrus phase, while it was otherwise always effective in males [226]. Finally, it has been reported that FAAH inhibition differentially affected males and females following single prolonged stress exposure, with males showing normalisation of hippocampal, prefrontal, and amygdala CB1 receptor expression following FAAH inhibition, whereas females only showed prefrontal and amygdala normalisation [227]. These studies show evidence that there are sex differences in the effects of endocannabinoid signalling on PTSD-like symptomology and behaviours, though the exact details of these differences are still emerging.

#### 3.3.3. Discrepant Literature and the Difference Between Long- and Short-Term Outcomes

While most research has been encouraging, it should be noted that not all studies have agreed with the above literature. Mayer et al. [228] found that the long-term (eight-day) effects of predator scent stress were not affected by THC administration post-stress, which only suppressed HPA activity and anxious behaviours short-term. In contrast, the administration of a CB1 antagonist was reported to normalise HPA activity and anxious behaviours after eight days post-stress [228]. This study contrasts with similar studies where it has often been assumed that the positive short-term effects of augmenting the endocannabinoid system post-stress (e.g., observed with favourable acute biological and behavioural changes) is an outcome measure that is ultimately relevant to the treatment of PTSD [229]. Indeed, other studies have found differences in short- compared to long-term effects of endocannabinoid modulation on PTSD-like symptoms. For example, Danan et al. [230] found that, immediately following stress, rats displaying PTSD-like behaviours had significantly elevated 2-AG levels in cerebral spinal fluid. However, after eight days, hippocampal AEA and 2-AG levels, as well as hypothalamic 2-AG levels, were significantly reduced in rats displaying PTSD-like symptomology [230].

Some studies have, though, reported that acute endocannabinoid modulation during or post-stress may reduce long-term fearful behaviours. Kondev, Morgan, Najeed, Winters, Kingsley, Marnett, and Patel [174] found that increasing 2-AG during predator scent stress acutely increased stress behaviours, but long-term was associated with a reduced stress response compared to controls. This effect was replicated in a separate study [231], and, interestingly, the effect of 2-AG was found to be CB2-, rather than CB1-, dependent [232], suggesting a potential divergence between AEA and 2-AG mechanisms in recovery from fear responses.

Finally, some studies have outright found no effect of cannabinoids on PTSD-like symptomology. For example, Huffstetler et al. [233] found that CBD increased anxious behaviours in the elevated plus maze and in the light–dark box tests, whereas Liu et al. [234] found no effect of CBD on anxious behaviour during the elevated plus maze. Moreover, THC is known to be anxiogenic at moderate to high doses [235]. Therefore, not all studies have provided support for reduction in PTSD-like symptomology via the endocannabinoid system.

#### 3.3.4. Subsection Summary

In summary, this section provided a broad but concise overview of the relationship between endocannabinoid signalling and preclinical models of PTSD. Overall, this body of literature provides evidence that the endocannabinoid system is involved in the development of PTSD-like symptoms, including fear memories, and are related to the extinction of fear memories. While these findings have important clinical implications, nuances and caveats have emerged, including age- and sex-specific effects, the importance of brain region locality and dose timing in determining the directionality of effects, and, in some cases, questions about the long-term efficacy of augmenting endocannabinoid signalling directly during or following trauma. In the next section, the role of endocannabinoids in translational (i.e., human) research will be comprehensively reviewed.

## 4. Understanding the Role of Endocannabinoid Signalling in PTSD in Humans

While preclinical literature has presented a reasonably consistent narrative on the role of endocannabinoid signalling in fear and PTSD-like symptoms, the translational step to understanding endocannabinoids in human PTSD has been an essential piece of the research puzzle. This section will comprehensively review human research studies testing the link between endocannabinoid signalling and fear conditioning, and between endocannabinoid signalling and real-life PTSD symptomology.

### 4.1. Human Tests of Endocannabinoids During Stress

While a succinct theory has been developed for endocannabinoid regulation of the stress response in animal models, human research on endocannabinoid activation following stress has been mixed [236]. The first study to examine human serum endocannabinoid responses to stress found that 2-AG increased immediately following psychosocial stress, while OEA and PEA (but not AEA) reduced after 30 min [237]. Likewise, hemodynamic stress was found to result in higher 2-AG levels, though this change occurred 45 min after stress induction rather than immediately [238]. Ney et al. [239] also found a small but significant increase in salivary 2-AG concentrations following a combined psychosocial and physical stressor, though no changes were observed to AEA. Meanwhile, Dlugos et al. [240] reported the finding that AEA serum concentrations increased following psychosocial stress. Several other recent studies have similarly reported increases in AEA following stress [241,242], though other research has found no significant effect of stress on AEA levels or 2-AG [239,241,242,243]. When subjected to meta-analysis, participants undergoing psychosocial stress show no overall significant changes to AEA or 2-AG [236]. However, there have only been a small number of studies conducted on this topic and all have had modest sample sizes, used different stress induction techniques, and collected different biomatrices from varying groups of participants, some with and without mental disorders.

### 4.2. Human Models of Fear Conditioning

#### 4.2.1. Seminal Fear Conditioning Endocannabinoids Research in Humans

**Figure 2:**
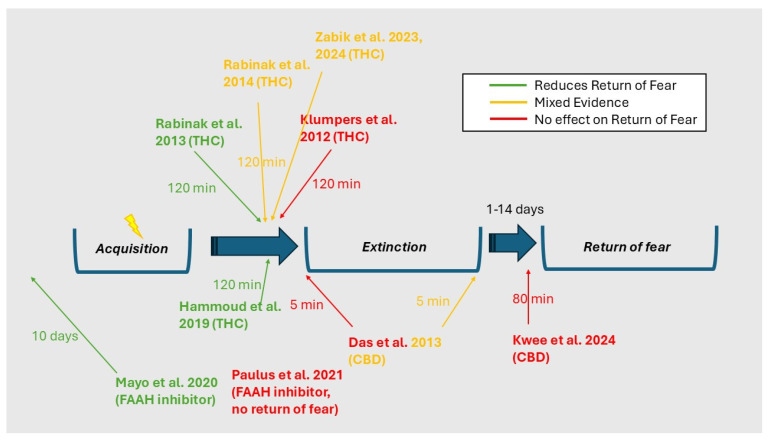
Effectiveness of cannabinoids on return of fear and the relationship to timing of cannabinoid administration. CBD = cannabidiol, FAAH = fatty acid amide hydrolase, THC = delta9-tetrahydrocannabinol. Das et al. 2013 [251], Hammoud et al. 2019 [254], Klumpers et al. 2012 [252], Kwee et al. 2024 [255], Mayo et al. 2020 [241], Paulus et al. 2021 [256], Rabinak et al. 2013 [250], Rabinak et al. 2014 [249], Zabik et al. 2024 [257].

Numerous studies over the past 15 years have tested how the endocannabinoid system might be involved in fear conditioning in humans [27,218,244,245,246,247]. The first such study was conducted by Heitland et al. [248], who tested whether the cannabinoid receptor (CB1) polymorphisms rs1049353 and rs2180619 were involved in the acquisition and extinction of conditioned fear. The authors reported that, while rs1049353 was not associated with either acquisition or extinction, A/A homozygotes of rs2180619 had impaired extinction learning, as measured by fear-potentiated startle [248]. In the years that followed, several studies assessed the effect of THC and CBD on fear conditioning [249,250,251,252], though the results were mixed (Figure 2). While Rabinak, Angstadt, Sripada, Abelson, Liberzon, Milad, and Phan [250] found that THC given prior to extinction learning improved 24-h extinction retention as measured by skin conductance, this effect was not replicated by Klumpers, Denys, Kenemans, Grillon, van der Aart, and Baas [252], who found no effect of THC on skin conductance or fear-potentiated startle in extinction testing. Similarly, Das, Kamboj, Ramadas, Yogan, Gupta, Redman, Curran, and Morgan [251] found no effect of CBD on skin conductance response, though did report that the subjectively rated consolidation of extinction learning was reduced in participants receiving CBD during extinction learning. Finally, while Rabinak, Angstadt, Lyons, Mori, Milad, Liberzon, and Phan [249] found that key extinction neural circuitry (hippocampus, ventromedial prefrontal cortex) was more active in a THC group during extinction retention testing compared to a placebo control, there were no differences in skin conductance responses between the THC and placebo groups. Based on this literature, early assessments of endocannabinoid involvement in human fear conditioning were not yet compelling, though some emerging trends were beginning to appear [253].

#### 4.2.2. Emphasis on the FAAH Polymorphism in Fear Conditioning

During this time, attention was beginning to pivot towards a single-nucleotide polymorphism located on the *FAAH* gene, rs324420. This polymorphism is involved in the differential expression of the FAAH enzyme, which is involved in the hydrolysis of AEA [258]. The presence of the minor (A) allele at rs324420 is associated with decreased FAAH activity and consequently higher AEA concentrations [259,260]. The first examination of rs324420 in fear conditioning found that participants with the minor A allele showed significantly enhanced fear extinction learning, as measured by skin conductance responses [261]. At the same time, evidence from other non-fear conditioning research was also beginning to suggest that, in humans, attention to threatening stimuli (and other measures of fear) were correlated with the rs324420 polymorphism [156,262,263,264], and the results similarly suggested that the A allele of the FAAH rs324420 polymorphism was associated with reduced fear-like behaviour.

Since then, research has increasingly focused on the importance of rs324420 on human fear conditioning, with a particular emphasis on how AEA might be centrally involved in regulation of these memory processes. Mayo et al. [265] reported further evidence that the presence of the minor A allele was associated with significantly enhanced fear extinction learning and extinction retention, as measured by fear-potentiated startle. Ney et al. [266] was able to partially replicate this effect, but only when AEA plasma levels moderated the analysis, and this study also reported differential rs324420 effects depending on PTSD symptom severity. Other studies that have tested rs324420 and blood AEA levels have been less consistent, with Zabik et al. [267] and Spohrs et al. [268] finding no genotyping effects on physiological or behavioural measures of fear conditioning. Despite these null findings, these and other studies have found that minor A allele carriers of rs324420 had higher brain activity in fear extinction circuitry during extinction learning [268,269] and extinction retention [267,270]. It was separately reported that AEA, but not 2-AG, mediated the effect of exercise on subjective threat expectancy ratings following extinction learning [271]. Moreover, hair levels of AEA and 2-AG have been reported to be associated with fear learning and the return of fear [272,273], though these studies found that higher 2-AG and lower AEA were associated with improved fear extinction.

The importance of AEA in human fear conditioning has resulted in increasing interest in the molecule as a potential way of improving exposure therapy outcomes (which rely on extinction learning) for PTSD [17,274]. One proposed method to achieve this is via pharmacological FAAH inhibition [150,275,276]. Mayo, Asratian, Lindé, Morena, Haataja, Hammar, Augier, Hill, and Heilig [241] trialled this approach in healthy participants using the FAAH inhibitor PF-04457845, which was administered ten days prior to fear extinction in a two-day, placebo-controlled conditioning paradigm. FAAH inhibition was reported to significantly improve performance on the extinction retention task was reported to result in a tenfold increase in circulating AEA levels [241]. However, Paulus et al. [256] found no effect of acute FAAH inhibition on fear acquisition or extinction learning, suggesting that longer-term dosing of the drug may be required to observe effects on conditioning.

#### 4.2.3. THC Administration During Fear Conditioning

Similar to studies of the FAAH polymorphism rs324420, studies that have administered THC prior to, or during, extinction learning have generally reported diminished amygdala responses and enhanced activation and/or coupling between higher-order brain regions involved in extinction (such as the ventromedial prefrontal cortex) during extinction retention tests [249,254,277,278]. Moreover, the effects of THC on brain activity measured during return of fear appear to be dose-dependent, with higher THC doses (10 mg) increasing prefrontal cortex and hippocampal activity longer term compared to lower THC doses (5 mg) [257]. Although there is some consistency in fear conditioning designs administering THC, a recently study found no evidence for enhanced fear re-extinction by CBD, though threat expectancy during retention, but not skin conductance or fear-potentiated startle responses, were reduced in the CBD (compared to placebo) group [255]. 

#### 4.2.4. Endocannabinoids During the Trauma Film Paradigm

While the fear conditioning paradigm has been explored extensively in the endocannabinoid field, to our knowledge only two studies have assessed whether endocannabinoids are associated with intrusive memories stemming from exposure to the trauma film paradigm. O’Donohue et al. [279] found that salivary AEA moderated the stress-induced effects of cortisol on the frequency of intrusive memories of traumatic imagery, though Ney, Cooper, Lam, Moffitt, Nichols, Mayo, and Lipp [272] found no evidence of a relationship between hair levels of AEA, 2-AG, OEA, or PEA and intrusive memories during a trauma film study. Future research is needed to explore this relationship more closely and in a wider range of biomatrices.

#### 4.2.5. Subsection Summary

In summary, the past decade has seen a large increase in the number of human studies examining the effect of cannabinoids and endocannabinoids on experimental fear conditioning. The improving and more widespread technology to measure endocannabinoids in human biomatrices [280] has partially facilitated this expansion of research, though it has largely been driven by significant optimism in the research community about this topic. Although early studies were mixed, more recent studies have mostly replicated animal literature, which suggests that higher levels of AEA (and, in some cases, 2-AG) may be associated with improved retention of extinction learning, which is the experimental analogue for exposure learning during therapy. Cannabinoid administration has similarly been somewhat mixed, though studies have reliably suggested that neural activity in circuitry believed to contribute to fear extinction memory are improved by concurrent THC delivery.

### 4.3. Association Between PTSD and Endocannabinoid Signalling in Humans

#### 4.3.1. Circulating Endocannabinoids and PTSD Symptomology

The relationship between endocannabinoid signalling and PTSD symptomology has been examined more directly in an increasing number of human studies. Firstly, the relationship between measurements of circulating endocannabinoids and PTSD symptoms has been tested. The first such study was conducted in survivors of the World Trade Centre attacks. This study found that survivors that met diagnostic criteria for PTSD had significantly reduced plasma levels of 2-AG and that less intrusive memory symptoms were associated with significantly higher AEA levels [281]. Similarly, Wilker et al. [282] reported that reduced hair levels of OEA were observed in veterans with PTSD compared to those without PTSD, and Bergunde et al. [283] found that lower hair levels of AEA (but not 2-AG) were associated with higher childbirth-related posttraumatic stress symptoms 2-months post-partum. In a positron emission tomography study, Neumeister et al. [284] similarly found that participants with PTSD had significantly reduced plasma levels of AEA compared to controls and displayed evidence of higher CB1 expression in the brain. In contrast, Hauer et al. [285] found that AEA, 2-AG, and OEA were all significantly increased in a small sample (*n* = 9) of patients with PTSD, compared to controls, and Schaefer et al. [286] found that serum OEA was higher in participants with PTSD, though no differences were found for AEA and 2-AG. Similarly, Croissant et al. [287] found no association between hair AEA, 2-AG, or OEA with PTSD symptomology in unaccompanied refugee minors, and Coccaro et al. [288], Leen et al. [289], Crombie et al. [290], and Ney, Matthews, Hsu, Zuj, Nicholson, Steward, Nichols, Graham, Harrison, Bruno, and Felmingham [266] found no baseline difference in AEA or 2-AG between subjects with and without trauma-related disorders. These studies therefore did not provide consistency in the direction of endocannabinoid status following PTSD diagnosis.

#### 4.3.2. FAAH Polymorphism and PTSD Symptomology

More recent studies have also tested the association between rs324420 polymorphism and PTSD symptoms. However, these studies unfortunately do not add clarity to the directionality of endocannabinoid effects on real-world PTSD. Both Marusak et al. [291] and deRoon-Cassini et al. [292] found that higher AEA levels and the presence of the rs324420 minor A allele were associated with higher PTSD symptoms. This effect would not be predicted by the fear conditioning literature [17]. In a group of female participants with PTSD, it was also found that carriers of the minor A allele showed overall higher activation of brain areas in the frontoparietal network during a fear acquisition and extinction task, suggesting enhanced executive functioning in these participants compared to CC homozygotes [269]. Marusak et al. [293] additionally reported that fractional anisotropy in the left fornix and left parahippocampal cingulum was associated with trauma exposure in youth. This study reported that the A minor allele of rs324420 was associated with lower fractional anisotropy in the left fornix and left parahippocampal cingulum, potentially indicating a reduced neural response to trauma when compared to CC homozygotes [293]. Moreover, Lazary et al. [294] found that the A minor allele of rs324420 was associated with higher depressive and anxiety symptoms in participants with histories of childhood trauma. Conversely, a large study (N = 4811 youths) found no effects of the rs324420 polymorphism on neural or anxiety responses to threatening faces [295], though this study did report that AA homozygotes had significantly lower symptoms of depression compared to A/C and CC carriers.

#### 4.3.3. Relationship Between Exercise, Endocannabinoids, and PTSD

Studies have also reported that, while exercise acutely affects endocannabinoid levels in circulation [296,297,298,299], exercise-induced changes in endocannabinoid levels may differ between participants with PTSD and healthy controls. While Botsford et al. [300] and Crombie, Leitzelar, Brellenthin, Hillard, and Koltyn [242] did not find any differences in blood levels of AEA and 2-AG at baseline, stress- and exercise-induced changes in 2-AG were only observed in the healthy control group in both of these studies. Crombie et al. [301] also found that the beneficial effects of exercise on mood, anxiety, and fear ratings in an uncontrollable threat task were correlated with exercise-induced increases in 2-AG (but not AEA) in both participants with and without PTSD.

#### 4.3.4. Endocannabinoids During Therapy and During Experimentation in Patients with PTSD 

Several studies have tested the endocannabinoid response during therapy and the effect of cannabinoids on therapeutic outcomes in clinical trials. However, again, the results are mixed. While Leen, de Weijer, van Rooij, Kennis, Baas, and Geuze [289] found no difference between veterans with PTSD and trauma-exposed controls in basal endocannabinoid plasma levels, measurements post-trauma exposed therapy showed that higher AEA and 2-AG were associated with higher anxiety, arousal, and general distress in patients with PTSD. By comparison, Rabinak et al. [302] found that THC had a beneficial effect on participants with PTSD who were completing a threat processing paradigm. Specifically, this study reported that THC administration (compared to placebo) was associated with reduced threat-related amygdala reactivity, higher medial prefrontal cortex activation during threat, and elevated functional coupling between the medial prefrontal cortex and amygdala [302]. Similarly, Pacitto et al. [303] found that the administration of THC to participants with PTSD reduced negative affect during a cognitive reappraisal task, an effect that was also associated with the normalisation of the angular gyrus response and increased dorsomedial prefrontal cortex response during the task.

#### 4.3.5. Trials and Retrospective Studies

Outside of experimental designs, cannabinoid administration has been associated with mostly beneficial effects during trials and retrospective studies. The first such study, Fraser [304], examined the medical records of PTSD patients who had been prescribed THC for the treatment of nightmares. This study found that THC significantly reduced PTSD-related nightmares in these patients [304]. Subsequent research, including a randomised controlled trial [305], found further evidence that THC is effective in reducing nightmares and some waking symptoms in patients with PTSD [306,307,308,309,310,311,312,313]. Multiple case studies have also reported beneficial effects of cannabinoids on waking and sleep-related PTSD symptoms [314,315,316,317,318], though the first randomised controlled clinical trial of different cannabinoid formulations for the treatment of PTSD found no difference to placebo [319]. Similarly, a matched case–control in veterans found no effect of cannabis use on PTSD symptoms [320] and, using a prospective longitudinal design, Metrik et al. [321] reported that higher intrusive memory symptomology was associated with cannabis use in patients with PTSD. Finally, a recent study found that CBD, a non-psychoactive constitute of cannabis, reduced cognitive impairment scores in participants with PTSD following recall of a traumatic event, though no differences between CBD and placebo were observed on a range of other factors such as alertness, anxiety, discomfort, or physiological data [322].

#### 4.3.6. Population-Based Cross-Sectional Studies

Population-based cross-sectional research is also mixed on whether cannabinoids are beneficial for PTSD. For example, while some research suggests that cannabis use is associated with worse PTSD symptoms and higher levels of comorbid mental health symptoms [321,323,324,325,326,327,328,329], other studies have found that cannabis use is correlated with reduced PTSD symptoms and comorbidities [330,331,332]. Other research has suggested that not only is cannabis use more frequent in PTSD [333,334], but there is also evidence that trauma reminders increase cannabis craving in participants with high levels of PTSD [335,336], suggesting the development of maladaptive dependence in certain patients, particularly those with comorbid cannabis use disorder [337,338]. This is especially important given that cannabis use disorder can be extremely debilitating for persons exposed to trauma and with PTSD [339,340]. However, some longitudinal research suggests that the relationship between adopting cannabis use and trauma exposure can be bidirectional [341].

#### 4.3.7. Subsection Summary

In summary, the available literature examining the relationship between endocannabinoid signalling and PTSD in humans is inconsistent, despite the relatively large number of studies conducted. There is mixed evidence of whether baseline endocannabinoid levels differ from healthy controls; however, one study that examined the long-term trajectory of endocannabinoid levels post-trauma may provide some context for why these findings differ so dramatically. Jayan et al. [342] measured AEA and 2-AG serum levels both immediately following trauma exposure and 6 months after trauma exposure in a group of hospitalised participants. It was found that mental health symptoms (dysphoria and high comorbidity) were associated with elevated AEA levels in the short term, but reduced AEA levels compared to resilient controls after 6 months [342]. This suggests that endocannabinoid tone may change over time as mental health symptoms worsen in individuals who develop PTSD. This possibility requires further testing. The available research is also inconclusive as to whether cannabinoids are beneficial in the treatment of PTSD. Although early research was optimistic, more recent studies have been mixed. The following section will summarise these discrepancies and provide a putative hypothesis for the conditions under which cannabinoids may be effectively used in PTSD treatment.

## 5. The Current Hypothesis, Challenges, and Future Directions

As has been reviewed in Section 3 and Section 4, there is an emerging hypothesis in preclinical research, and to a lesser extent in human literature, suggesting that the endocannabinoid system may be involved in PTSD symptomology and memory-related processes such as fear conditioning. While there is mixed evidence for this position, many researchers in the field are optimistic that cannabinoid augmentation may be an effective treatment for PTSD symptoms, if used under the right conditions. This section will briefly outline this hypothesis in the field before describing some challenges that the research community faces in translating this understanding into a useful clinical tool for the real-world treatment of PTSD.

### 5.1. The Current Hypothesis and Approaches to Treatment

Currently, the research literature potentially supports two treatment approaches for cannabinoid interventions in PTSD. Firstly, a large proportion of preclinical research, and some human research, suggests that the inhibition of the FAAH enzyme is a potentially useful strategy for reducing PTSD symptoms [17]. The reduction of symptoms through CB1 agonism following from FAAH inhibition has been hypothesised to be due to enhancement of extinction learning [343], though alternative mechanisms for its therapeutic effects have also been proposed, such as the reduction of stress and hyperarousal symptoms [9].

The second treatment approach that has been favoured by the research literature is the direct agonism of CB1 receptors by THC or similar cannabis plant constituents such as cannabinol. While this approach is mostly supported by preclinical literature [23], the research community generally leans towards FAAH inhibition as the preferred strategy for cannabinoid augmentation of treatment for PTSD. The reason for this preference is that THC, as a CB1 agonist, will produce psychoactive side effects, tolerance, potential dependency, and other physiological side effects at doses relevant to therapy [344,345]. On the other hand, psychoactive side effects and dependency of FAAH inhibition are less likely to occur [17,275,346]. This is because FAAH inhibition has not been documented to produce pleasurable or psychoactive effects that are sought by recreational or dependent users of THC.

While many studies have tested the above approaches, some researchers believe that CBD will additionally have beneficial effects for PTSD treatment [30,213]. However, this approach is not universally supported as although CBD produces anxiolytic and extinction-enhancing effects via the 5-HT1A and CB1 receptors, respectively, the overall biological mechanisms of CBD are not well characterised, and it is established that CBD does not interact directly with CB1 receptors [118,347,348,349]. While non-interaction with CB1 is likely the main reason that CBD does not produce psychoactive effects, the preclinical literature has firmly established that CB1 activity is critical for cannabinoid-mediated benefits to extinction learning and other PTSD-relevant processes [9]. Therefore, many researchers in the cannabinoid field believe that while CBD may have anxiolytic effects in many scenarios [350,351,352,353,354], it is unlikely to be as effective as THC or FAAH inhibition in reducing PTSD symptoms, which are strongly associated with memory dysfunction. Despite this, the fact that CBD does not produce CB1-mediated psychotropic effects means that it may be a safer alternative to THC, should research show significant benefit for PTSD-like symptoms in the future.

### 5.2. Challenges and Future Directions

#### 5.2.1. Future Directions in Experimental Research

While the research hypothesis above may seem compelling, significantly more research is needed to understand why such a large number of discrepant findings have been reported in the field, particularly in human literature. Firstly, human studies have not consistently identified a direction of effect for endocannabinoid activity following acute psychosocial stress [236]. Unfortunately, this field has high heterogeneity both in the direction and magnitude of effects, but also in how studies were conducted. For instance, not all of the studies in this field have used exclusively psychosocial stressors, and studies have varied substantially both in the participant populations and collected biomatrices [236]. Future studies will need to significantly increase sample sizes and systematically test across these variables, whilst also controlling for factors such as time of day [355] and the biological sex [356] and age [184] of the participants. It would also be timely to assess biological responses to stress in humans across a range of biomatrices in each new study, given the uncertainty about the origin of serum or plasma endocannabinoids [357], and the enormous distance between the level of invasiveness of biomatrix collection in animal compared to human studies.

Such factors must also be controlled in studies comparing endocannabinoid concentrations between clinical populations and healthy participants, which have also been somewhat inconsistent across studies [236]. Future research needs to establish what effect the FAAH rs324420 polymorphism has on the propensity to develop psychiatric disorders. This is because while experimental studies purport that the minor A allele should reduce the risk of PTSD-like symptomology [156,265], many studies from patient populations suggest that the A allele may present a risk factor for psychiatric disorders [291,292,293,294]. Ney, Matthews, Hsu, Zuj, Nicholson, Steward, Nichols, Graham, Harrison, Bruno, and Felmingham [266] identified a potential explanation for this: participants with PTSD showed poorer fear conditioning if they had the A allele of rs324420, which was an effect that was not present in trauma-exposed or healthy control participants. Therefore, it is possible that participants with PTSD or other psychiatric disorders may respond more severely to tasks that invoke stress if they have the A allele, even if it is a protective factor more generally. Future studies will need to examine all of these discrepancies using a mixture of clinical and healthy participant groups.

#### 5.2.2. Clinical Trials

The above approaches to treatment are based primarily on preclinical research, with human translational research only beginning to emerge. As was reviewed in Section 4, human research is mixed. Therefore, the first challenge of the PTSD–cannabinoid research field is the adequate and comprehensive translation of preclinical findings to human subjects. Research must move towards clinical trials, as even our best experimental models in PTSD research, such as fear conditioning, have frequent questions raised about their validity [73,74,79,358,359,360,361]. It must be established that the modulation of the endocannabinoid system affects memory processes that are relevant to PTSD in humans and reduces PTSD symptomology in patients with the disorder.

Unfortunately, the first placebo-controlled randomised clinical trial showed no efficacy of cannabinoids in reducing PTSD symptoms [319]. Based on mixed evidence within the human fear conditioning literature and cross-sectional data, this result may have been predicted. However, we have previously argued that there are reasons why this trial may have failed [20,362]. Firstly, from the preclinical literature, we know that the modulation of the endocannabinoid system is most effective at reducing PTSD symptoms if it is conducted concurrently with extinction training [182]. This means that patients should only be prescribed the drug during or before exposure therapy sessions, where endocannabinoid augmentation will facilitate successful extinction learning that occurs during the therapy session. In contrast, Bonn-Miller, Sisley, Riggs, Yazar-Klosinski, Wang, Loflin, Shechet, Hennigan, Matthews, Emerson, and Doblin [319] did not require participants to take part in any psychological therapy in conjunction with their cannabinoid intervention. This result therefore supports the preclinical literature where cannabinoid augmentation did not reduce PTSD-like behaviours if the animal was not undergoing concurrent extinction training [182].

This evidence implies that future clinical trials need to be carefully designed so that cannabinoids are treated as adjunctive therapy alongside routine exposure or similar psychological interventions. Moreover, cannabinoids should be trialled in larger experimental studies to ascertain whether they are effective in improving experimental extinction learning, given that the existing human literature is underpowered and inconsistent. It is interesting to note that meta-analysis suggests that trauma-focused therapy works equally well regardless of cannabis use in patients with PTSD [363,364]. While this might sound foreboding for a cannabinoid adjunctive approach to therapy, the studies included in these meta-analyses did not control cannabinoid timing relative to therapy. Regardless, these meta-analyses further reinforce the idea that timing of cannabinoid intervention is a critical factor to consider, as it is increasingly clear that the prescription of cannabinoid products will not necessarily improve treatment outcomes even if psychological therapy is additionally completed. It is very likely that cannabinoids need to be used with precise timing relative to psychological therapy to have the strongest benefits.

#### 5.2.3. Contextual and Logistical Challenges

There are many other logistical challenges to adopting cannabinoids as treatments for PTSD. Firstly, cannabinoids such as THC are restricted in most countries and, for the most part, are still not legalised even for medicinal purposes. In countries where cannabinoids have become legalised for medical purposes, the approved indications are typically limited. For example, in the United Kingdom, cannabinoids can legally be prescribed for severe and rare forms of epilepsy, nausea caused by chemotherapy, and multiple sclerosis. Similarly, while cannabis is now legal in many parts of the United States of America, it is not approved for indications beyond those approved in the United Kingdom. The accessibility of medicinal cannabinoids is also severely restricted. For example, in Australia, even though cannabis can be accessed for medical purposes for non-approved conditions (such as chronic pain or PTSD), patients must apply through elongated pathways that can delay treatment. Therefore, the cannabinoid field described in the current article is seriously limited in short- and medium-term applicability to real-world patients given the status of legality and access of cannabinoid products. Cannabinoid use also conflicts with drug-free driving laws in many countries [365]. In Australia, for example, THC cannot legally be detectable in a person’s saliva if this person wishes to operate a motor vehicle, even if the THC is medically prescribed [366,367,368]. Not only does this pose issues for clinical practice, but it prevents clinical trials from being feasible within the Australian (and many other countries’) landscape.

Other logistical issues also exist. For example, cannabinoids (such as THC) have high first pass metabolism that varies significantly within individuals, between individuals, and depending on factors such as sex and body mass index [199,369,370,371,372,373]. Therefore, establishing accurate dosing for a patient may be difficult, especially if small differences in AEA, CBD, or THC availability produce large differences in therapeutic effects. Given the issues with variable metabolism of cannabinoids, clinical monitoring methods are important to ensure accurate dosing. However, methods of clinical monitoring for cannabinoids are still in development and existing techniques have recently come under scrutiny. For example, meta-regression suggests that impairment is not correlated with THC-related oral and blood biomarkers [374], and commercially available oral THC test kits have recently been found to inaccurately detect recent use of oral THC [375]. Moreover, in plasma, CBD can be detected for several weeks after digestion of a moderate to high dose [376], though for both THC and CBD, there is no known quantitative reference point for impairment [377,378]. Based on this research, effective clinical monitoring of cannabinoid therapies is a challenge that will need to be met in future studies.

#### 5.2.4. Risks of Cannabinoid Use in PTSD

The risks of cannabinoids are well characterised and present an additional complicating factor to prospective use for indications such as PTSD. Firstly, THC has psychoactive effects, which may adversely affect patients with PTSD, which are a vulnerable clinical population [362]. THC has the potential to produce anxiogenic effects if administered at the wrong dose [379,380,381,382], which may cause more harm to PTSD patients during therapy. CBD has also been documented to produce anxiogenic effects at higher doses [353,354]. Multiple studies also show evidence that use of cannabinoids can increase apathy and amotivation [379,383], episodes of paranoia and panic [379], and potentially cause psychosis, especially in patients with a predisposition to psychotic disorders [384,385]. Long-term cannabinoid use is also associated with tolerance towards the drug [386,387], dependency [388], and impairments in cognition [389] and can result in cannabis use disorder, which is characterised by severe impacts to mental health and wellbeing [390]. These psychiatric side effects have important implications for use within vulnerable patient populations such as those with PTSD, who are particularly susceptible to comorbid substance abuse [363,391,392]. Cannabinoids such as THC and CBD also have a wide range of physical side effects such as sedation, impaired psychomotor function dizziness, dry mouth, cardiovascular problems, and gastrointestinal disturbances, which can become chronic in some cases [393,394,395,396,397,398].

Finally, cannabinoids have variable times of onset, which are dependent on the route of administration, product formulation, and individual metabolism [399,400,401,402,403,404,405]. Therefore, significant research is required to understand how it is possible to precisely time and safely administer cannabinoid interventions during PTSD therapy [406]. This issue has multiple implications, including therapist time, patient monitoring, and potentially increased therapy session duration to align with other psychoactive interventions for PTSD.

### 5.3. Section Summary

In summary, although much of the scientific picture is becoming clear, the logistics for how cannabinoid interventions for PTSD will be administered in the future are far from resolved. Future studies urgently need to establish the best therapy combinations to progress to clinical trials as well as the best product specifications including route of administration, product formulations, individual dosing, dosing timing, and therapy schedules. Studies will also need to navigate the complex and potentially far-reaching adverse effects that may threaten the safety of cannabinoids for use amongst PTSD populations. This body of work promises to be as substantial as all of the preceding work in this field; yet, the research community has not yet pivoted towards answering these important questions.

## 6. Summary and Conclusions

This article reviewed the role of endocannabinoids in fear conditioning, PTSD-like symptomology, and PTSD. PTSD is a disorder that is often resistant to treatment; therefore, more effective treatment methods are actively sought by the research community. The modulation of the endocannabinoid system has been proposed as one such solution. As outlined in this review, endocannabinoid signalling has been identified as potentially critical to fear conditioning processes, which are believed to be an important part in the development of and recovery from PTSD. Moreover, the preclinical literature suggests that endocannabinoids may also be involved in some other PTSD-like behaviours following trauma exposure.

While the preclinical literature is reasonably consistent, emerging human research has shown a more complex picture. Studies testing physiological responses during fear conditioning have not consistently replicated the animal literature, and cross-sectional data concerning cannabinoid efficacy in recovery from PTSD is mixed. This article suggested multiple future directions for human research that may help to build a more consistent and effective treatment approach to cannabinoid-adjunctive therapy for PTSD. These suggestions include clinical trials and experimental research focusing specifically on issues such as medicinal timing, the route of administration, and product formulation, all of which may impact treatment efficacy. Moreover, this review poses the question as to how variable metabolism of cannabinoid medicines will be managed in a clinical setting, especially given that small differences in cannabinoid availability may have differential effects on treatment outcomes [257]. Future studies need to carefully consider the factors outlined in this article before the proposed interventions become a feasible option for clinicians.

## Abbreviations

The following abbreviations are used in this manuscript:

2-AG	2-arachidonoyl glycerol
AEA	Arachidonoyl ethanolamide
CB1	Cannabinoid Receptor 1
CB2	Cannabinoid Receptor 2
CBD	Cannabidiol
CS	Conditioned stimulus
DAGL	Diacylglycerol lipase
FAAH	Fatty acid amide hydrolase
HPA	Hypothalamic–pituitary–adrenal
MAGL	Monoacylglycerol lipase
OEA	Oleoylethanolamide
PEA	Palmitoylethanolamide
PTSD	Posttraumatic stress disorder
THC	Delta9-tetrahydrocannabinol
US	Unconditioned stimulus
